# Supplementary material for: Distributed coding of duration in rodent prefrontal cortex during time reproduction
Source: eLife. 2021 Dec 23;10:e71612. doi: 10.7554/eLife.71612 (PMC8786316; doi:10.7554/eLife.71612)
Supplement: Transparent reporting form [file elife-71612-transrepform1.pdf]

eLife
Westbrook Centre
Milton Road
Cambridge CB4 1YG, UK
P 01223 855340
W elifesciences.org
T @elife

eLife's transparent reporting form

We encourage authors to provide detailed information *within their submission* to facilitate the interpretation and replication of experiments. Authors can upload supporting documentation to indicate the use of appropriate reporting guidelines for health-related research (see EQUATOR Network), life science research (see the BioSharing Information Resource), or the ARRIVE guidelines for reporting work involving animal research. Where applicable, authors should refer to any relevant reporting standards documents in this form.

If you have any questions, please consult our Journal Policies and/or contact us: editorial@elifesciences.org.

Sample-size estimation

- You should state whether an appropriate sample size was computed when the study was being designed
- You should state the statistical method of sample size computation and any required assumptions
- If no explicit power analysis was used, you should describe how you decided what sample (replicate) size (number) to use

Please outline where this information can be found within the submission (e.g., sections or figure legends), or explain why this information doesn't apply to your submission:

Sample sizes are given in the Methods section "Animals" and the Results. As no effect sizes could be defined beforehand, no sample-size calculations were performed.
The number of three animals is low for a rodent study. The behavioral paradigm requires extensive training (about 2 months for each animal, detailed in Methods "Behavioral training and testing"), similar to comparable studies in primates that also use low animal numbers. Therefore, we decided to compensate for the low number of animals by performing larger numbers of experimental sessions in each animal (38, 32, 31 sessions, reported e.g. in Fig. 1E).
Recording of different neurons across sessions was ensured by adjustment of the electrode placement (see Methods "Recording procedures"). This allowed recording of 1766 mPFC neurons (see Methods "Analysis of electrophysiological data").

Replicates

- You should report how often each experiment was performed
- You should include a definition of biological versus technical replication
- The data obtained should be provided and sufficient information should be provided to indicate the number of independent biological and/or technical replicates
- If you encountered any outliers, you should describe how these were handled
- Criteria for exclusion/inclusion of data should be clearly stated
- High-throughput sequence data should be uploaded before submission, with a private link for reviewers provided (these are available from both GEO and ArrayExpress)

Please outline where this information can be found within the submission (e.g., sections or figure legends), or explain why this information doesn't apply to your submission:

We performed a total of 105 experimental sessions (38, 32, 31 sessions for each animal, respectively), with more than 50 trials on average (Fig. 1–Figure supplement 2A).
Behavioral and electrophysiological data were acquired in well trained animals only (see Methods "Behavioral training and testing"). No data from training sessions is included in the paper, apart from Fig. 1–Figure supplement 2D.
Quality of spike sorting and stability was assessed as described in Methods "Spike sorting" (see Figure 2–Figure supplement 1C for an example).

Statistical reporting

- Statistical analysis methods should be described and justified
- Raw data should be presented in figures whenever informative to do so (typically when N per group is less than 10)
- For each experiment, you should identify the statistical tests used, exact values of N, definitions of center, methods of multiple test correction, and dispersion and precision measures (e.g., mean, median, SD, SEM, confidence intervals; and, for the major substantive results, a measure of effect size (e.g., Pearson's r, Cohen's d)
- Report exact p-values wherever possible alongside the summary statistics and 95% confidence intervals. These should be reported for all key questions and not only when the p-value is less than 0.05.

Please outline where this information can be found within the submission (e.g., sections or figure legends), or explain why this information doesn't apply to your submission:

Statistical analysis methods included in multiple places within the paper and are specified in Methods. If exact p-values are not reported significance is indicated asterisks (see Methods "Additional notes on data analysis").

(For large datasets, or papers with a very large number of statistical tests, you may upload a single table file with tests, Ns, etc., with reference to sections in the manuscript.)

Group allocation

- Indicate how samples were allocated into experimental groups (in the case of clinical studies, please specify allocation to treatment method); if randomization was used, please also state if restricted randomization was applied
- Indicate if masking was used during group allocation, data collection and/or data analysis

Please outline where this information can be found within the submission (e.g., sections or figure legends), or explain why this information doesn't apply to your submission:

Responses of all recorded neurons are usually analyzed together. If subgroups were allocated, it is described within the respective part of the Results section and Figures.

eLife Sciences Publications, Ltd is a limited liability non-profit non-stock corporation incorporated in the State of Delaware, USA, with company number 5030732, and is registered in the UK with company number FC030576 and branch number BR015634 at the address Westbrook Centre, Milton Road, Cambridge CB4 1YG.

Additional data files ("source data")

- We encourage you to upload relevant additional data files, such as numerical data that are represented as a graph in a figure, or as a summary table
- Where provided, these should be in the most useful format, and they can be uploaded as "Source data" files linked to a main figure or table
- Include model definition files including the full list of parameters used
- Include code used for data analysis (e.g., R, MatLab)
- Avoid stating that data files are "available upon request"

Please indicate the figures or tables for which source data files have been provided:

Figure 1–source data 1. Source data for Figure 1E&F and Figure 1–Figure Supplement 2A-C. Figure 1–source data 2. Source data for Figure 1–Figure Supplement 2C1&C2 and Figure 1–Figure Supplement 3.
Figure 1–source data 3. Source data for Figure 1–Figure Supplement 2D.

Figure 3–source data 1. SDF source data for Figure 3A&B and all upcoming analyses and figures based on SDFs. Figure 3–source code 1. Source code to load Figure 3–source data 1.

Raw data is available at Henke J, Bunk D, von Werder D, Häusler S, Flanagin V, Thurley K (2021) Data for Distributed coding of duration in rodent prefrontal cortex during time reproduction. G-Node. https://doi.org/10.12751/g-node.tarvrs.

eLife Sciences Publications, Ltd is a limited liability non-profit non-stock corporation incorporated in the State of Delaware, USA, with company number 5030732, and is registered in the UK with company number FC030576 and branch number BR015634 at the address Westbrook Centre, Milton Road, Cambridge CB4 1YG.
